# Physical Activity and Mental Health in Undergraduate Students

**DOI:** 10.3390/ijerph20010195

**Published:** 2022-12-23

**Authors:** Gabriel Rodríguez-Romo, Jorge Acebes-Sánchez, Sonia García-Merino, María Garrido-Muñoz, Cecilia Blanco-García, Ignacio Diez-Vega

**Affiliations:** 1 Faculty of Physical Activity and Sport Sciences (INEF), Universidad Politécnica de Madrid, 28040 Madrid, Spain; 2 Faculty of Health Sciences, Universidad Francisco de Vitoria (UFV), 28223 Madrid, Spain; 3 Faculty of Psychology, Universidad Francisco de Vitoria (UFV), 28223 Madrid, Spain; 4 Departamento de Enfermería y Fisioterapia, Facultad de Ciencias de la Salud, Universidad de León, 24007 León, Spain

**Keywords:** mental health, physical activity, sports, exercise, undergraduate students

## Abstract

Most research support positive relationships between physical activity and mental health. However, possible moderating variables of these relationships have also been identified, such as age, gender, level of physical activity, and the scope of physical activity. This study aimed to analyze the relationships between physical activity and mental health levels in undergraduate students, assessing whether these associations can change depending on the level of physical activity (low, medium, or high) and the setting (occupational, commuting, or leisure time physical activity) in which it was performed. A descriptive and cross-sectional study was conducted. The sample comprised 847 undergraduate students. Physical activity and mental health were measured by the Global Physical Activity Questionnaire (GPAQv2) and the General Health Questionnaire (GHQ-12). We found relationships between students’ physical activity level and their mental health status. The higher the total physical activity, the better their mental health scores. High levels of commuting and leisure time physical activity is also associated with better mental health, while only moderate levels of occupational physical activity are associated with better mental health status. Regarding the possible associations between physical activity and vulnerability to mental health problems, with the fully adjusted regression model, leisure time and occupational physical activity remain protective of a poor state of mental health. Leisure time physical activity, performed at a high level, and moderate occupational physical activity seems to be the best combination of physical activity to reduce students’ vulnerability to potential mental health problems.

## 1. Introduction

The World Health Organization [1] defines mental health (MH) as a “state of well-being in which an individual realizes his or her abilities, can cope with the normal stresses of life, can work productively and can make a contribution to his or her community”. Positive MH is not only the absence of negative symptoms but also the behaviors that make people have healthy living habits to maintain good MH [2]. The impairment of MH has been perceived with increasing concern, especially in geographic areas with higher rates of economic development [3].

One of the habits related to better MH is being physically active. High physical activity (PA) is related to mental well-being and social well-being [4,5,6,7,8], in addition to physical well-being [9,10]. Higher levels of PA are positively correlated with general well-being and negatively correlated with symptoms of depression and anxiety [11]. Low PA levels are associated with an increased prevalence of anxiety [12]. Similarly, a meta-analysis of 92 studies concluded that PA has a significant medium effect in reducing symptoms of depression and a low effect in reducing symptoms of anxiety [13]. On the other hand, two meta-analyses conclude that PA can confer protection against the emergence of depression [14,15].

University students are in a time of transition in their lives. For many, this involves many changes in their living habits as they move from a structured environment (high school) to a relatively unstructured environment (university) [16]. This period can be one of the most stressful in a person’s life [17]. In fact, according to İlhan et al. [18], university life involves highly challenging responsibilities, new and unfamiliar situations, and more complex academic tasks. It means that university students are vulnerable and have a high prevalence of MH disorders [19], with anxiety and depression being the most common [17]. Furthermore, the transition from high school to college means a drop in students’ overall PA levels [20,21,22,23].

A study that reports the first stage of the WHO World Mental Health International College Student project, with 13,984 first-year full-time students, showed that 31% screened positive for at least one 12-month MH disorder [24]. Another similar research developed by Alonso et al. [25], with first-year students from 19 universities in eight countries, shows that one in three (31.4%) students have suffered some mental disorder in the first year of university. In one in five (20.4%) cases, these disorders have affected their social, personal, work, and academic life, assuming severe disability. These data do not improve throughout the university years, as shown by research carried out with undergraduate students at UK universities [26]. This study finds that 42.3% of the participants had experienced a serious personal, emotional, behavioral, or MH problem for which they needed professional help. In addition, they point out that students in the third year of the course are more likely to report past psychological issues. Research developed by Blasco et al. [27] with Spanish university students from five universities exposes that around 10% of Spanish students declare to have had suicidal thoughts in the last 12 months.

University students who meet PA recommendations are more likely to report good MH [28]. Research with university students shows that moderate and high levels of PA are inversely related to anxiety and depression scores [17,29]. These data are also supported by Herbert [30], who found that university students who perform regular PA correlate negatively with depression, anxiety, and stress and positively correlate with the quality of life [30]. This relationship occurs in a dose-response manner, as pointed out by Grasdalsmoen et al. [31], who note that physical exercise is negatively associated with all measures of MH problems and suicidality.

Most research support positive relationships between PA and MH. However, possible moderating variables of these relationships have also been identified, such as age [32,33], gender [34], PA level, and scope of realization [35]. Likewise, different investigations propose the need to provide updated and local information that can be useful for defining policies and strategies to promote PA [36].

Considering all the above, the following research questions were proposed: Is PA related to a better MH in undergraduate students from Madrid? Is that relation depending on the type or level of PA? In order to answer these research questions, this study aims to analyze the relationships between PA and MH levels, assessing whether these associations can change depending on the level of PA (low, medium, or high) and the setting (occupation, commuting, or leisure time) in which it was performed, in university students in Madrid, Spain.

## 2. Materials and Methods

### 2.1. Sample and Procedure

A descriptive and cross-sectional study was conducted using an online questionnaire designed for the investigation as a data collection instrument.

The study population was university students in the region of Madrid. Participants had to be undergraduate students at any public or private university in the Madrid region. Online undergraduate or master’s degree students were excluded. Considering an odds ratio of 0.81 [37], a Variance Inflation Factor of 0.1, an alpha risk of 0.05, and a beta risk of 0.2, a sample size of 859 participants was required to detect significant associations between poor mental health and physical activity practice. Disproportionate stratified sampling was used according to the type of university (public or private) and the academic discipline of the student (social and juridical sciences; engineering and architecture; arts and humanities; health sciences; physical and life sciences).

A total of 893 surveys were collected. After reviewing and refining the surveys received (eliminating contradictory answers and empty or incomplete questionnaires), the final sample comprised 847 undergraduate students from public and private universities in Madrid.

Lecturers from all universities (six public and seven private) in Madrid were contacted by email. The contact information was collected on the internet. Some lecturers from all contacted universities decided to collaborate voluntarily. Participants were contacted through their lecturers, who sent them a Google Forms Questionnaire. Before participation in the study, informed consent was obtained for the completion of the survey. Participation was voluntary and confidential. Sociodemographic (gender and age) and academic information were collected to examine the possible moderating effect of these variables relating to the associations between PA and MH. Data collection was from January 2022 to May 2022.

### 2.2. Measures

#### 2.2.1. Physical Activity

PA was measured by the Global Physical Activity Questionnaire (GPAQv2), which contains 16 questions and captures information about PA in a typical week [38,39]. This questionnaire provides information about the intensity (moderate or intense), frequency (days in a typical week), and duration (hours and minutes in a typical day) of PA performed across its three domains: (i) occupational (OPA): related to a paid or unpaid job, studies, housework, or job search (item example: “In a typical week, on how many days do you do vigorous-intensity activities as part of your work?”); (ii) commuting-related (CPA): walking or cycling (item example: “Do you walk or use a bicycle (pedal cycle) for at least 10 minutes continuously to get to and from places?”); and (iii) leisure-time (LTPA) (item example: “In a typical week, on how many days do you do vigorous-intensity sports, fitness or recreational (leisure) activities?”). It also contains a question assessing the time (in minutes) spent sitting or reclining during a typical day.

The GPAQ derives from the International Physical Activity Questionnaire (IPAQ), which has been validated and widely used to assess PA patterns [40,41]. GPAQ shows good reliability and validity in assessing PA patterns (Kappa 0.67 to 0.73; Spearman’s rho 0.67 to 0.81). This investigation used the Spanish version without modifying the content or the original text. The GPAQ protocol was strictly followed for data collection and processing [39].

From the duration (minutes), intensity (moderate, vigorous), and frequency (days per week) of physical activities performed in a typical week, PA-related energy expenditure was calculated according to the guidelines of the questionnaire’s data treatment protocol [39,42,43,44]. Total PA (sum of OPA, CPA, and LTPA) was classified into three levels (high, moderate, and low) according to the time spent on PA per day in a typical week, the number of days on which this PA was performed and the intensity of this PA [39] (see Table 1).

Based on the PA recommendations, the cut-off points were used [45]. Thus, participants with low PA levels were defined as insufficiently active, i.e., they performed no PA or did not fulfill the minimum recommendations for PA to have a health benefit. In contrast, those assigned to the moderate and high PA levels were sufficiently active individuals, i.e., those who fulfilled or exceeded these recommendations.

To calculate the levels (high, moderate, and low) of PA in each area or domain (occupational, commuting, and leisure time) the same criteria were followed as those outlined for total PA, although only PA carried out in the area in question was considered.

#### 2.2.2. Mental Health

The Spanish version of the General Health Questionnaire (GHQ-12), developed by Goldberg and Williams [46], was used to assess MH. It is a self-administered screening instrument that aims to detect psychological morbidity and possible cases of psychiatric disorders in contexts such as primary care or the general population [47]. The GHQ-12 is one of the most widely used validated screening instruments [48] and is widely recommended for use in health surveys [49].

In different studies, the GHQ-12 shows good validity (Area under the receiver operating characteristic curve of 0.88) and reliability (Cronbach’s alpha of 0.82) [50], as well as in the Spanish population [51]. Its structure, which covers the four fundamental psychiatric areas (depression, anxiety, social inadequacy, and hypochondriasis), is efficient in the identification of cases in different population groups [47]. Specifically, it has been shown that the GHQ-12 can be used effectively to assess psychological well-being and detect non-psychotic psychiatric problems in the Spanish population [51].

The GHQ-12 consists of 12 propositions—six related to the inability to perform normal functions (item example: “Have your worries made you lose a lot of sleep?”) and six related to the presence of new distressing experiences (item example: “Have you lost confidence in yourself?”). Each item describes a symptom related to psychological distress, anxiety, and depression. Each item has four possible answers presented on a 4-option Likert-type scale (“not at all”, “no more than usual”, “somewhat more than usual” and “much more than usual”). The responses obtained are quantified using the GHQ score, which offers the best psychometric properties for the detection of vulnerability to mental disorders [47,50]. With the GHQ score, the Likert-type scale response options are transformed to a dichotomous-type scale (0–0-1–1). When the item is worded in a way that expresses a symptom, the responses “not at all” and “no more than usual” take the value 0, and the responses “somewhat more than usual” and “much more than usual” take the value 1. In contrast, if the item is worded positively, the values for rating each category are reversed. The total score is the sum of all the values obtained in each item; it is understood that as the scores increase, the level of MH decreases. Thus, we have a scale with a maximum value of 12 points and a minimum of 0. According to studies that validate the GHQ-12 against standardized psychiatric interviews [46], those scores that reach or exceed 4 points are considered “cases” or threshold of the pathological state.

### 2.3. Data Analysis

Frequencies, percentages, medians, and quartiles were used as descriptive statistics.

Crosstabs and Chi-square test was used to explore the differences in the distribution of the presence or absence of MH along the domains and levels of PA, gender, and age.

Kruskal–Wallis and Bonferroni’s post hoc corrections were used to evaluate the differences in the MH score of the participants based on the level and domain of PA.

Finally, two logistic binomial models were calculated. Previously, the basic assumptions were verified. The first model (crude model) assesses the bivariate associations of each level and domain of PA with the presence/absence of MH. The second model (adjusted model) studies the same associations but in a multivariate way. The odds ratio (OR) and their respective confidence intervals (CI 95%) were informed as measures of effect size. All statistical analyzes were performed with SPSS v.26, setting a significance level of *p* = 0.05.

## 3. Results

### 3.1. Characteristics of the Sample

The sample was composed of 847 undergraduate students (21.56 ± 4.73 years: 21.53 ± 4.32 years for men and 21.57 ± 4.88 years for women). The mean MH score, measured through the GHQ-12 questionnaire, was 3.79 ± 3.31, with a median score of 3. The characteristics and distribution of the sample are presented in Table 2, comparing the distribution of participants with an adequate level of mental health and a poor state of mental health throughout the study variables.

### 3.2. Mental Health at Different Levels and Domains of Physical Activity

Table 3 reports the median value of MH (GHQ12) at the different levels and domains of PA practice. It can be seen how people who perform a high level of total PA have a lower score (M = 2; RIQ = 4) in the GHQ12 questionnaire compared to those who achieve low (M = 4; RIQ = 5) or moderate (M = 3.5; RIQ = 5) levels of total PA (χ^2^ (2) = 25.64; *p* < 0.001), which suggests a better state of mental health in people who practice more PA. In addition, differences were observed in all the PA domains. Participants who achieve the highest level of LTPA and CPA receive a lower score on the GHQ12 questionnaire than participants who perform low LTPA (χ^2^ (2) = 32.2; *p* < 0.001) and low CPA (χ^2^ (2) = 7.91; *p* = 0.019), which again suggests a better state of mental health among the highest practitioners of PA. Finally, in the occupational domain, participants who reach moderate levels of OPA obtain better scores than those with high or low levels of OPA (χ^2^ (2) = 8.45; *p* = 0.015). These results show that people who have a low level of PA on the move, such as using the car, have higher levels of mental illness. Likewise, those who need to commute with high levels of PA also show higher levels of mental illness.

### 3.3. Associations between Mental Health and Levels and Domains of Physical Activity

Table 4 presents logistic regression models to evaluate the association between the presence of a mental illness (GHQ12 ≥ 4) and the practice of PA.

In the first model (bivariate model), we can observe how all PA dimensions are associated with the state of mental illness. Accumulate a high level of PA in LTPA (OR= 0.47; CI95%= 0.35–0.63), as in CPA (OR= 0.63; CI95%= 0.42–0.93) or perform a moderate level of OPA (OR= 0.4; CI95%= 0.19–0.83), protects from a mental illness state.

These results are modified in the second model (multivariate-adjusted model), highlighting the role of the high level of LTPA (OR= 0.51; CI95% = 0.37–0.7) and a moderate level of OPA (OR= 0.47; CI95%= 0.22–0.99), which are the only domains that remain protective of mental illness when the total adjustment of the model is carried out. This highlights the importance of performing occupational activities with an adequate level of PA and, above all, of performing a high level of PA during free time to avoid mental illness status.

## 4. Discussion

This research aimed to analyze the relationships between PA and MH levels in university students, assessing whether these associations can change depending on the level of PA (low, medium, or high) and the setting in which it was performed (OPA, LTPA, or CPA).

Our results confirmed the presence of relationships between PA and MH in undergraduate students. It also found a high prevalence of MH problems among students, as well as high percentages of physical inactivity. PA and MH relationships vary according to the level of PA and the domain in which it is performed. Likewise, when analyzing the associations between PA levels and domains and the likelihood of MH disorders, the LTPA performed at a high level, as well as moderate OPA, are shown to have the most robust association in terms of reducing students’ vulnerability to potential MH problems.

A high percentage of the undergraduate students (45.5%) who participated in this study suffer from some type of mental disorder (GHQ12 score ≥ 4), with the incidence being significantly higher in females (48.4%) than in males (38.1%). These results are in line with the research carried out by Qureshi et al. [52], who found in a sample of undergraduate students from Pakistan that a large number of the students (42.6%) had mild depression. Likewise, Üner et al. [53], in an investigation of undergraduate students from Turkey, showed that 56.8% of students had a GHQ12 score ≥ 4. This research concludes that the most stressful events were not being able to study the career or university they wanted, losing a family member, or breaking up with their partner. According to Auerbach et al. [24], colleges across the world are contending with rising rates of mental disorders. However, this research, which presents the initial results from the first stage of the WHO World Mental Health International College Student project (19 colleges across eight countries), showed a lower incidence of common lifetime disorders (31%). In any case, the results are high, and the undergraduate students are suffering. In this vein, Pereira et a.l. [26] showed that 42.3% of university students from the UK had experienced a serious personal, emotional, behavioral, or MH problem for which they needed professional help. Even more serious are the recent results from five Spanish Universities, which reported that around 10% of Spanish students declare to have had suicidal thoughts in the last 12 months [27]. On the other hand, our results revealed that almost a quarter (24.6%) of university students are insufficiently active, considering their total PA. This percentage rises to 60% when only LTPA is considered, i.e., that which individuals decide to do on a completely voluntary basis. These results are similar to previous studies [54,55]. For instance, 24% of U.S. undergraduate students do not achieve recommended PA levels [54].

The results of the present study show positive relationships between the PA level of university students and their MH status. These associations are found both when considering total PA and when analyzing the different domains in which it takes place (OPA, LTPA, and CPA). Indeed, the higher the level of total PA performed by university students, the better their MH scores and MH status. Likewise, higher levels of CPA and LTPA are associated with better MH. In the case of OPA, associations with better MH status are only found when PA is performed at a moderate level compared to low or high levels.

As in the present study, previous work has also shown positive relationships between PA and MH [6,56]. Likewise, Chu et al. [57] showed that higher levels of LTPA were associated with lower psychological distress. Several investigations have been carried out to explain these results. Attempts have been made to analyze the biochemical causality of the effect of PA on MH without definitive results [58]. In contrast, evidence has been found that social support and engagement around PA could largely explain the observed effects on mood changes [35]. In this sense, our study may provide a further argument for a sociological explanation of these mechanisms. The subjective perception of being an active person is positively associated with physical fitness [59]. LTPA is a voluntary act that responds to different needs. One is the free choice to undertake a leisure activity, which we consciously recognize as being of interest in improving our health. On the other hand, this activity is linked to a social environment that is different from the usual one, which gives us access to a new leisure environment, both in terms of the type of relationships and the objectives.

Likewise, our results of CPA are similar that found in different studies [2,60,61,62]. It is important to be mentioned that the existing evidence supports the idea that cycling is better than walking for the maintenance and improvement of MH in workers [60]. Martin et a.l. [61] showed that people who travel actively had significantly better MH than those who use public transport or car travel. Some approaches could be interesting to improve the MH of employees or students. Thus, as mentioned by Tamminen et al. [2], urban planning to develop active travel for pedestrians and cyclists and incentives at work, such as showers and storage facilities, could contribute to improving the mental well-being of employees or, in this case, undergraduate students.

Despite other studies [2,62], we found that moderate levels of OPA are associated with better MH. However, Mason et al. (2016) mentioned that OPA contributes to increasing overall PA and will therefore be beneficial and should be considered. In contrast, White et al. [63], in their meta-analysis, point out that LTPA provides a distraction from stress, but OPA does not; indeed, work itself may be a source of stress. In this sense, our results could be interesting to the extent that an occupation with no PA (sedentary), as well as a job with too much PA (exhaust), could worsen MH.

Regarding the possible associations between PA and vulnerability to MH problems, the results obtained show that high levels of total PA, LTPA, and CPA, as well as moderate levels of OPA, are associated with a lower probability of suffering from MH disorders. However, with the fully adjusted regression model, these associations only hold for LTPA and OPA, highlighting the greater strength of these associations. In this sense, students who engage in a high level of LTPA or a moderate level of OPA have a 47% and 51% lower risk, respectively, of suffering from MH problems than those who are insufficiently active during their leisure time. These results are partially consistent with previous research [6,57]. Chu et al. [57] stated that in the Asian population, higher levels of LTPA were consistently associated with less psychological distress. Rodríguez-Romo et al. [6], in an adult Spanish sample, point out that PA level during leisure time showed an inverse relationship with vulnerability to mental disorders. A meta-analysis developed by White et al. [63] supports our results, concluding that LTPA is likely to be the most effective method of preventing mental ill-health. However, Hamer et al. [64], in a general population sample, found that any form of daily PA was associated with a lower risk of experiencing MH problems. Likewise, they also found a dose-response pattern, with a greater reduction in risk for higher volume and/or intensity activities and stronger relationships when PA consisted of sport. On the other hand, an umbrella review of 23 health outcomes across 158 observational studies developed by Cillekens et al. [65] found better mental health for those who engage in high versus low OPA. However, this umbrella also detects that high OPA levels were positively related to depression and anxiety.

In summary, considering the high prevalence of MH problems among the students who participated in this study and their high rates of sedentary lifestyles, our findings highlight the need to promote PA among university students, both for its health benefits in general and its positive associations with MH. From a practical point of view, PA presents itself as a useful and cost-effective tool to protect university students from the onset of potential MH problems. In this sense, our results suggest that efforts should focus on the development of policies and action strategies aimed specifically at promoting LTPA in sufficient quantity and intensity to enable students to achieve high levels of PA in this domain. In this sense, university sports services could play a key role, with a wide, regular, and quality offer of sports facilities and activities. In addition, specific actions aimed at encouraging active cycling to universities, as well as on university campuses themselves, could also contribute to improving the physical activity levels and mental health of university students. On the other hand, concerning OPA, it may be interesting for universities to propose PA modules as cross-cutting opportunities to develop better PA levels while improving knowledge of better habits.

The present study is not without limitations. Firstly, its cross-sectional nature prevents us from establishing causal relationships between the variables analyzed. On the other hand, the lack of additional information on comorbidities could have affected the relationships found between PA and MH. Likewise, although assessing PA with self-report tools is more economical and feasible, and the GPAQ has been validated in different countries [42], there are objective PA assessments using pedometers and accelerometers that may be more accurate tools and avoid PA overestimation that often occurs in questionnaires [66,67]. However, we believe that the tools we have used for this research can be considered a strength. Among the most frequent criticisms of studies that have analyzed the possible relationships between PA and MH was the lack of reliability of the instruments used [34,68]. In the present study, reliable and valid tools were used to measure PA and MH (GPAQv2 and GHQ-12, respectively). Furthermore, despite the possible problems of GPAQv2 overestimation, this tool has made it possible to structure the PA according to levels (low, moderate, and high) and different domains (OPA, LTPA, and CPA). In our opinion, this last aspect is a relevant contribution compared to other previous studies, which, for the most part, have focused on analyzing the relationships between MH and total PA or, to a lesser extent, between MH and LTPA. Our results allow us to deepen our understanding of these relationships, distinguishing the contribution that PA, depending on its level and scope, may have on MH. In addition, the use of the GHQ-12 has made it possible to assess MH’s general state, as opposed to other studies focused exclusively on analyzing the possible relationships between PA and certain pathologies (i.e., depression and anxiety). The use of both questionnaires, GPAQv2 and GHQ-12, self-administered, has made it possible to reach a large sample of undergraduate students, with the advantage that this represents in terms of possible generalization of the results obtained. Although previous research on the relationships between MH and PA already exists, the results of this study provide updated and local information that can be very useful to define policies and strategies to promote PA among university students in the Community of Madrid.

## 5. Conclusions

The main conclusion drawn from the present study is that a high-level LTPA and a moderate-level OPA (considering its volume, frequency, and/or intensity) seem to be the best combination of PA to reduce students’ vulnerability to potential MH problems. Our results show the existence of relationships between students’ PA level and their MH status, both for the total PA and the PA performed in each of the domains analyzed (OPA, LTPA, and CPA). Thus, the higher the total PA, the better their MH scores. High levels of CPA and LTPA are also associated with better MH, while in the case of OPA, only moderate levels of OPA are associated with better MH status.

## Figures and Tables

**Table 1:** Total physical activity levels and classification criteria recommended by Global Physical Activity Questionnaire.

Level of Total Physical Activity	Physical Activity Thresholds
High	≥3 days of vigorous activity (at work and during recreation) in a typical week AND a total physical activity MET-minutes per week ≥1500. ≥7 days of vigorous activity (commuting, at work, and during recreation) in a typical week AND a total physical activity MET-minutes per week ≥3000.
Moderate	Does not achieve the above criteria but does achieve one of the next following: ≥3 days of vigorous activity (at work and during recreation) in a typical week, at least 20 min each day. ≥5 days of vigorous or moderate activity (commuting, at work, and during recreation) in a typical week, at least 30 min each day. ≥5 days of vigorous or moderate activities (commuting, at work, and during recreation) in a typical week, at least 600 MET-minutes per week of total physical activity.
Low	Do not achieve the high or moderate criteria.

**Table 2:** Sample characteristics and distribution of healthy (GHQ12 < 4) and unhealthy (GHQ12 ≥ 4) mental status.

	Total	Healthy	Unhealthy	*p*
Total	847	462 (54.5)	385 (45.5)	
Gender				
Male	239	148 (61.9) ^b^	91 (38.1) ^a^	0.009
Female	608	314 (51.6) ^a^	294 (48.4) ^b^	
Age				
18–19	281	147 (52.3)	134 (47.7)	0.611
20–24	464	262 (56.5)	202 (43.5)	
25–30	66	33 (50)	33 (50)	
>30	36	20 (55.6)	16 (44.4)	
Total PA				
Low	208	94 (45.2) ^b^	114 (54.8) ^a^	<0.001
Moderate	274	137 (50)	137 (50)	
High	365	231 (63.3) ^a^	134 (36.7) ^b^	
Occupational PA				
Low	754	404 (53.6)	350 (46.4)	0.039
Moderate	39	29 (74.4) ^a^	10 (25.6) ^b^	
High	54	29 (53.7)	25 (46.3)	
Leisure-time PA				
Low	508	244 (48) ^b^	264 (52) ^a^	<0.001
Moderate	50	26 (52)	24 (48)	
High	289	192 (66.4) ^a^	97 (33.6) ^b^	
Commuting PA				
Low	340	176 (51.8)	164 (48.2)	0.054
Moderate	363	195 (53.7)	168 (46.3)	
High	144	91 (63.2) ^a^	53 (36.8) ^b^	

Results are expressed as Frequency (percentage). PA: Physical Activity. ^a^ Post hoc comparisons: higher number of participants observed than expected. ^b^ Post hoc comparisons: fewer number of participants observed than expected.

**Table 3:** Scores of mental illness (GHQ12) according to levels and domains of PA.

	PA Level			
	Low ^a^	Moderate ^b^	High ^c^	*p*
Total PA	4 (2–7) ^c^	3,5 (1–6) ^c^	2 (1–5) ^a,b^	<0.001 *
Occupational PA	3 (1–6) ^b^	1 (0–4 ) ^a,c^	3 (1.75–5) ^b^	0.015 *
Leisure-time PA	4 (1–7) ^c^	3 (1–7)	2 (1–5) ^a^	<0.001 *
Commuting PA	3 (1–7) ^c^	3 (1–6)	2 (1–4.75) ^a^	0.019 *

Results are expressed as Median (Quartile 1–Quartile 3). Superscript letters reflect significant post hoc differences between categories in each domain. PA: Physical Activity; * *p* < *0*.05.

**Table 4:** Associations between PA and presenting an unhealthy state of mental illness (GHQ12 ≥ 4).

	Model 1		Model 2	
	OR	CI95%	OR	CI95%
Occupational PA				
Low	1		1	
Moderate	0.4 *	0.19–0.83	0.45 *	0.21–0.94
High	1.00	0.57–1.73	1.28	0.71–2.28
Leisure-time PA				
Low	1		1	
Moderate	0.85	0.48–1.53	0.91	0.51–1.64
High	0.47 *	0.35–0.63	0.49 *	0.35–0.67
Commuting PA				
Low	1		1	
Moderate	0.93	0.69–1.24	0.98	0.72–1.33
High	0.63 *	0.42–0.93	0.85	0.55–1.32

Model 1= Bivariate crude model; Model 2 = Physical activity domains and levels adjusted model; Odds Ratio (OR); CI (Confidence Interval); PA: Physical Activity; * *p* < 0.05.

## Data Availability

The datasets used and/or analyzed during the current study are available from the corresponding author on reasonable request.
